# Supplementary material for: H2O2 promotes trimming-induced tillering by regulating energy supply and redox status in bermudagrass
Source: PeerJ. 2024 Feb 29;12:e16985. doi: 10.7717/peerj.16985 (PMC10909351; doi:10.7717/peerj.16985)

**Supplementary Figure 1:** **Altered CKs degradation enzymes in stem nodes of bermudagrass after trimming.** We identified 15 *CKX* genes from the bermudagrass genome and tested their temporal response to trimming. Asterisks indicating significant differences between each trimming time and 0 h by *t*-test(*P*<0.05).
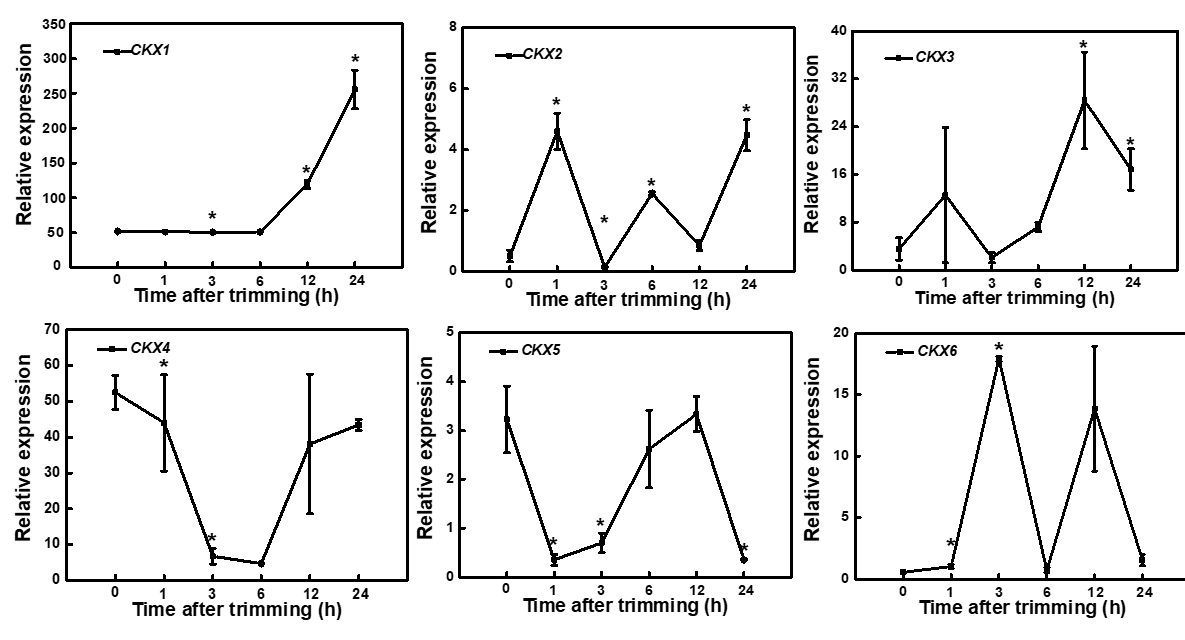


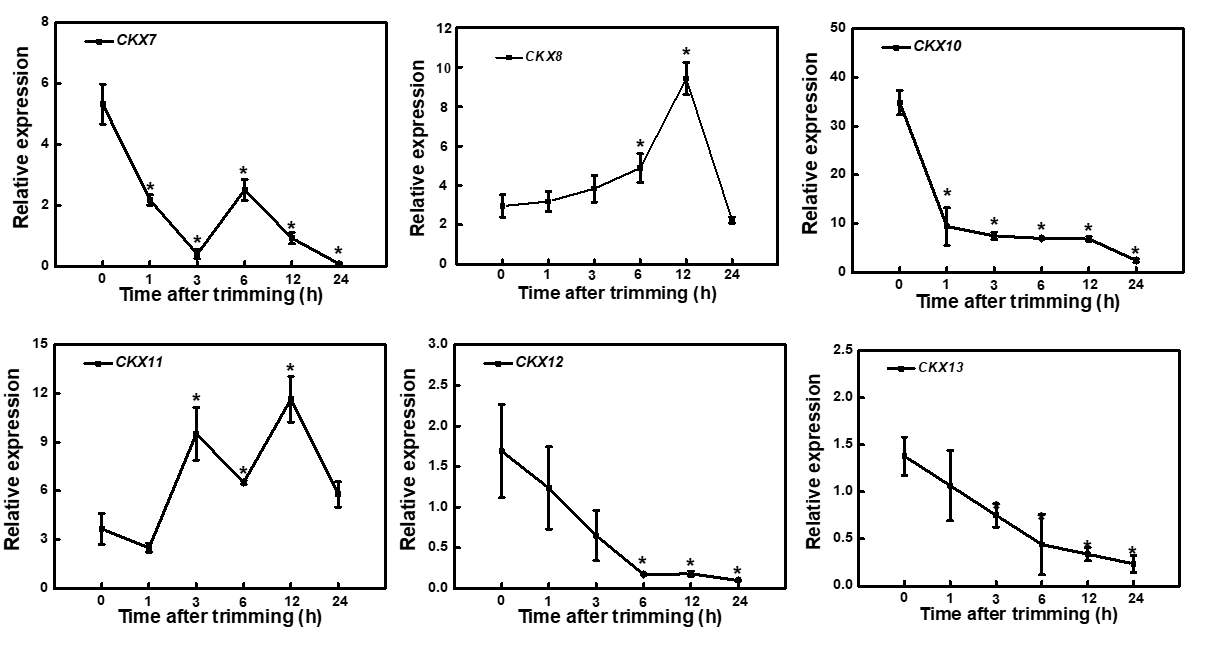


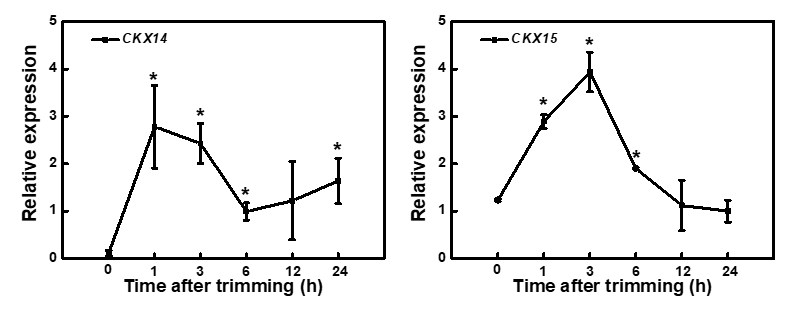

Supplement: Supplemental Information 1 — We identified 15 CKX genes from the bermudagrass genome (our unpublished data) and tested their temporal response to trimming. Asterisks indicating significant differences between each trimming time and 0 h by t-test (P < 0.05). [file peerj-12-16985-s001.docx]
