# Supplementary material for: H2O2 promotes trimming-induced tillering by regulating energy supply and redox status in bermudagrass
Source: PeerJ. 2024 Feb 29;12:e16985. doi: 10.7717/peerj.16985 (PMC10909351; doi:10.7717/peerj.16985)

**Supplementary Figure 2:** **Effect of exogenous treatments on bermudagrass tillering.** (a) Phenotype of the effect of exogenous 6-BA and sucrose on stolon tillering. (b) Effects of exogenous 6-BA and sucrose on the length of tiller buds at 1-5 nodes, with asterisks indicating significant differences between the intact control and treatment groups (6-BA, sucrose) as determined by One-Way ANOVA(*P*<0.05).


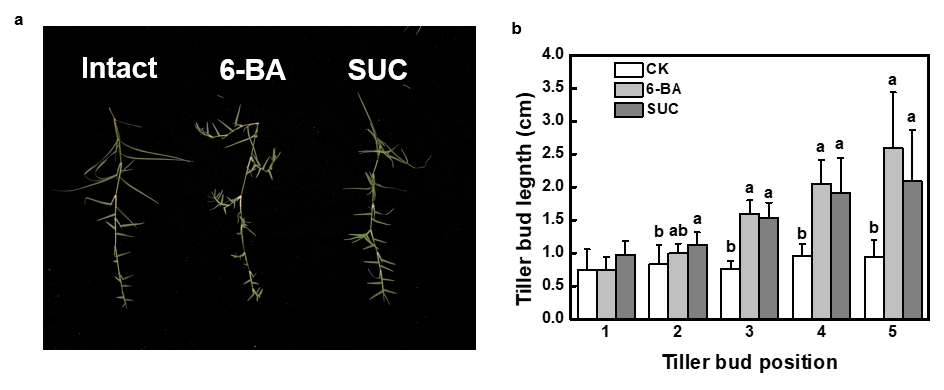

Supplement: Supplemental Information 2 — (A) Phenotype of the effect of exogenous 6-BA and sucrose on stolon tillering. (B) Effects of exogenous 6-BA and sucrose on the length of tiller buds at 1–5 nodes, with asterisks indicating significant differences between the intact control and treatment groups (6-BA, sucrose) as determined by One-Way ANOVA (P < 0.05). [file peerj-12-16985-s002.docx]
