# Supplementary material for: H2O2 promotes trimming-induced tillering by regulating energy supply and redox status in bermudagrass
Source: PeerJ. 2024 Feb 29;12:e16985. doi: 10.7717/peerj.16985 (PMC10909351; doi:10.7717/peerj.16985)
Supplement: Supplemental Information 3 [file peerj-12-16985-s003.docx]

**Supplementary Table.1：Primers used in real-time qPCR analysis**

| Gene | Forward primer | Reverse primer |
| --- | --- | --- |
| *Actin* | TCTGAAGGGTAAGTAGAGTAG | ACTCAGCACATTCCAGCAGAT |
| *TB1* | GACCGGCACAGCAAGATATG | TCGTCGGTCATGATCTCCTG |
| *IPT1* | GACGCCACCGAAGTGTTCAT | TGAGGGCTGAAACCATGCTG |
| *LOG1* | TGAGATGGCTCGGTTTGCTG | AGCCATCCACGTTCAGGAGA |
| *SPS* | AGGGTGGACCTCTTCACTCG | GCTCTCACCTCCTTCTCCGT |
