# Supplementary material for: H2O2 promotes trimming-induced tillering by regulating energy supply and redox status in bermudagrass
Source: PeerJ. 2024 Feb 29;12:e16985. doi: 10.7717/peerj.16985 (PMC10909351; doi:10.7717/peerj.16985)
Supplement: Supplemental Information 4 — Asterisks indicating significant differences between intact and trimmed samples by t-test, single asterisks denoting significance (P < 0.05), and double asterisks denoting high significance (P < 0.01). [file peerj-12-16985-s004.docx]

**Supplementary Table.2:** **Photosynthetic parameters extracted from OJIP transient curves**

Asterisks indicating significant differences between intact and trimmed samples by *t*-test, single asterisks denoting significance(*P*<0.05), and double asterisks denoting high significance(*P*<0.01).

|  | **Intact** | **6hTrimming** | **24hTrimming** | **Definitions** |  |
| --- | --- | --- | --- | --- | --- |
| **Data extracted from the recorded fluorescence transient OJIP** | | | | | |
| **F_0_** | 0.47±0.06 | 0.52±0.01 | 0.55±0.02 | Fluorescence at time 20 μs after onset of actinic illumination |  |
| **Fm** | 1.95±0.23 | 1.99±0.12 | 2.18±0.08 | Maximal recorded fluorescence intensity, at the peak P of OJIP |  |
| **Fv/Fm** | 0.76±0.01 | 0.74±0.01* | 0.75±0.00 | Reaction PSII reaction center light energy conversion efficiency, measured after 20 min of leaf dark adaptation |  |
| **F_k_** | 1.10±0.14 | 1.05±0.07 | 1.17±0.07 | Fluorescence value at 300 μs |  |
| **F_j_** | 1.30±0.16 | 1.26±0.08 | 1.42±0.06 | Fluorescence value at the J-step (2 ms) of OJIP |  |
| **Fluorescence parameters derived from the extracted data** | | | | | |
| **Vj** | 0.57±0.03 | 0.50±0.01* | 0.53±0.01 | Relative variable fluorescence at J step |  |
| **Vi** | 0.84±0.01 | 0.83±0.00 | 0.80±0.01 | Relative variable fluorescence at I step |  |
| **M0** | 1.69±0.02 | 1.43±0.06** | 1.52±0.14 | Approximated initial slope (in ms^−1^) of the fluorescence transient |  |
| **Quantum yields and efficiencies** | | | | | |
| **φP_0_** | 0.76±0.01 | 0.74±0.01* | 0.75±0.00 | Maximum quantum yield of primary photochemistry (at t = 0) |  |
| **Ψ_0_** | 0.44±0.00 | 0.50±0.01** | 0.47±0.01* | Efficiency/probability that an electron moves further than Q_A_- |  |
| **φE_0_** | 0.33±0.01 | 0.36±0.01** | 0.35±0.01 | Quantum yield of electron transport (at t = 0) |  |
| **φD_0_** | 0.24±0.01 | 0.26±0.01 | 0.25±0.00 | Quantum yield (at t = 0) of energy dissipation (at t = 0) |  |
| **φR_0_** | 0.12±0.01 | 0.13±0.00 | 0.15±0.01* | Quantum yield for reduction of end electron acceptors at the PSI acceptor side |  |
| **δR_0_** | 0.37±0.02 | 0.35±0.00 | 0.43±0.03* | Efficiency/probability with which an electron from the intersystem electron carriers moves to reduce end electron acceptors at the PSI acceptor side (RE) |  |
| **γ RC** | 0.20±0.00 | 0.21±0.01 | 0.21±0.01 | Probability that a PSII Chl molecule functions as RC |  |
| **Specific energy fluxes (per Q_A_-reducing PSII reaction center/RC)** | | | | | |
| **ABS/RC** | 2.30±0.03 | 2.09±0.10* | 2.13±0.15 | Absorption flux (of antenna Chls) per RC (at t=0) |  |
| **TR_0_/RC** | 3.02±0.02 | 2.83±0.11* | 2.84±0.20 | Trapping flux (leading to Q_A_ reduction) per RC (at t=0) |  |
| **ET_0_/RC** | 1.33±0.01 | 1.40±0.07 | 1.32±0.06 | Electron transport flux (further than Q_A_−) per RC (at t=0) |  |
| **ABS/RC** | 2.30±0.03 | 2.09±0.10* | 2.13±0.15 | Absorption flux (of antenna Chls) per RC (at t=0) |  |
| **TR_0_/RC** | 3.02±0.02 | 2.83±0.11* | 2.84±0.20 | Trapping flux (leading to Q_A_ reduction) per RC (at t=0) |  |
| **Performance indexes** | | | | | |
| **PI_ABS_** | 0.63±0.04 | 0.71±0.02* | 0.70±0.09 | Performance index (potential) for energy conservation from exciton to the reduction of intersystem electron acceptors |  |
| **PI_Total_** | 0.37±0.06 | 0.38±0.01 | 0.53±0.04* | Performance index (potential) for energy conservation from exciton to the reduction of PSI end acceptors |  |
| **PI_CS_** | 0.29±0.03 | 0.37±0.01** | 0.38±0.05* | Performance index on cross section basis |  |
