# Supplementary material for: H2O2 promotes trimming-induced tillering by regulating energy supply and redox status in bermudagrass
Source: PeerJ. 2024 Feb 29;12:e16985. doi: 10.7717/peerj.16985 (PMC10909351; doi:10.7717/peerj.16985)
Supplement: Supplemental Information 5 [file peerj-12-16985-s005.docx]

**Supplementary Table.3：*CKX* primers used in real-time qPCR analysis**

| Gene | Forward primer | Reverse primer |
| --- | --- | --- |
| *CKX1* | GAACTCTGCCCTCTTCTTCG | CGTAGTTCTTGGTGACCTCG |
| *CKX2* | GGAAGATGCTGACAAGGGAC | AAACACTTCCTCCTCCTCCA |
| *CKX3* | CATCATGTACCCGGTGAACC | TACTCCTTGTACCCGATCCC |
| *CKX4* | ACCATCCCAAGAACACCAAC | CTTCCATCCTGACCCGATTG |
| *CKX5* | CATCCTTGTCTACCCGCTTC | GAGCACGCTGAAGATGTACA |
| *CKX6* | GATGGAGAAGAAGGTGAGCG | GTAGCTCGGGAAGTAGGTCT |
| *CKX7* | CTACCTCTACCTCTCCGTGG | CGTAGTTCTTGGTGACCTCG |
| *CKX8* | GGAGGAGGAGGAAGTGTTCT | CAAGCAAGTGGGTACGAGAA |
| *CKX9* | GCCACAGATATCCAACGTGT | AGGTTGCTGTTCACGTAGAC |
| *CKX10* | CACCCATTTCCACTCTACGG | GAAGTCTGATGAAGCCTCCG |
| *CKX11* | AGGGCCTCATCCTCATCTAC | ATGCCGACCACATACATCAC |
| *CKX12* | GGTCAGTTCGGGGTCATAAC | AATGTAGTAGATTGCGCCGG |
| *CKX13* | CATCCTTGTCTACCCGCTTC | GAGCACGCTGAAGATGTACA |
| *CKX14* | CTGGATGGGGAGAAGAAGGT | CATGGGGTAGATGAGCATGG |
| *CKX15* | CCCACAGCATCGAACATACA | CTTCCACCTTGCCTCAAAGT |
