# Supplementary material for: H2O2 promotes trimming-induced tillering by regulating energy supply and redox status in bermudagrass
Source: PeerJ. 2024 Feb 29;12:e16985. doi: 10.7717/peerj.16985 (PMC10909351; doi:10.7717/peerj.16985)
Supplement: Supplemental Information 7 [file peerj-12-16985-s007.docx]

**EXPERIMENTAL DESIGN**

Definition of experimental and control groups

Control group: untrimming stolon nodes of bermudagrass.

Experimental group: trimming stolon nodes of bermudagrass.

Number within each group

Nodal sample was taken after 0, 1, 3, 6, 12, and 24 h upon trimming treatment.

Intact:0 h. Trimming:1, 3, 6, 12, 24 h. Three replicates per treatment.

From each of the first four nodes of bermudagrass stolons was taken after 24 h of trimming treatment.

Intact:1st, 2nd, 3rd, 4nd; Trimming: 1st, 2nd, 3rd ,4nd. ( Materials and Methods, Trimming treatment in manuscripts .)

**SAMPLE**

Description

Volume/mass of sample processed

When bermudagrass produces more stolons, stolon nodes as test samples were selected.

0.1g nodal was taken.

Processing procedure

Freeze samples in liquid nitrogen immediately after taken and stored at -80 ℃, then one day later RNA was extracted by grinding process.

**NUCLEIC ACID EXTRACTION**

Procedure and/or instrumentation

Total RNA was extracted using the Plant RNA Kit (Vazyme Biotech Co., Ltd).

Details of DNase or RNase treatment

The pipette tips and tubes were RNase-free.

Nucleic acid quantification

RNA concentration and quality were measured using a NanoDrop microspectrophotometer (Eppendorf).

Purity (A260/A280): A260/A280>1.9

RNA integrity method/instrument

RNA electrophoresis


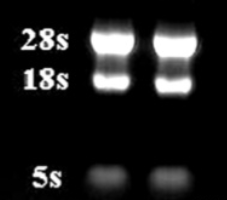


**REVERSE TRANSCRIPTION**

RNA was reverse transcribed to obtain cDNA with HiScript II 1st Strand cDNA Synthesis Kit (Vazyme Biotech Co., Ltd).

| RNase-free ddH_2_O | 7µL |
| --- | --- |
| 4 x gDNA wiper Mix | 4µL |
| RNA | 5µL |

Pipette gun blowing and mixing.42 ℃ 2 min.

| 5 x HiScript III qRT SuperMix | 4µL |
| --- | --- |
| First Step Reaction Solution | 16µL |

Pipette gun blowing and mixing.

| 37 ℃ | 15 min |
| --- | --- |
| 85 ℃ | 5 sec |

Storage conditions of cDNA

cDNA is stored at -4 ℃.

**qPCR TARGET INFORMATION**

Sequence accession number

*BRC1*(Gene ID: 821386 NC_003074.8).

*LOG1*(Gene ID: 817376 NC_003071.7).

*IPT1* (Gene ID: 843175 NC_003070.9).

*SPS* (Gene ID: 832150 NC_003076.8).

Amplicon length(100-150bp).

**qPCR OLIGONUCLEOTIDES**

qPCR oligonucleotides: the primer sequences are in Supplemental Data S1.

**qPCR PROTOCOL**

cDNA was employed as a template for qRT-PCR (Thermo, America) to detect gene expression using SYBR qPCR Master Mix (Vazyme Biotech Co., Ltd).

| 2 x Taq Pro universal SYBR qPCR Master Mix | | | 10 µL | |
| --- | --- | --- | --- | --- |
| Primer(F) | | | 0.5 µL | |
| Primer(R) | | | 0.5 µL | |
| Template DNA/cDNA | | | 2 µL | |
| ddH_2_O | | | 7 µL | |
| Amplification Protocol | | |  | |
| cycle step | temperature | time | | cycle number |
| predegeneration | 95 ℃ | 5 min | | 1 |
| denaturation | 95 ℃ | 10 sec | | 40 |
| annealing | 60 ℃ | 30 sec | | 40 |

Manufacturer of plates/tubes and catalog number

Yeasen Biotechnology(shanghai).

CAT:83555ES03 LOT:MO2022120836

**qPCR VALIDATION**

Specificity (gel, sequence, melt, or digest)

The dissolution curve is single peaked.


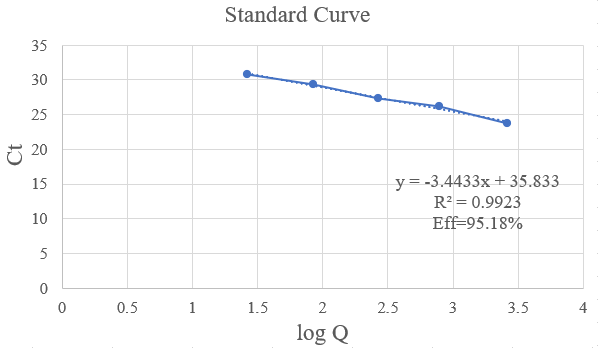


**DATA ANALYSIS**

qPCR analysis program was offered by Thermo, America. The technique was repeated three times for each sample.

qPCR dates are in raw date, significance by t-test(P<0.05), SPSS16.0.
